# *Rhodotorula* fungemia: A series of four cases

**DOI:** 10.1016/j.mmcr.2026.100823

**Published:** 2026-09-18

**Authors:** Amira Dallali, Aicha Kallel, Sarra Cheikhrouhou, Sonia Marouen, Nour Ben Abdeljelil, Chokri Kaddour, Kalthoum Kallel

**Affiliations:** a Department of Parasitology-Mycology, La Rabta Hospital, Tunis, Tunisia; b Department of Hematology and Transplant, National Bone Marrow Transplant Center , Tunis, Tunisia; c Department of Intensive Care, National Institute of Neurology Mongi-Ben Hamida, Tunis, Tunisia; d Faculty of Medicine of Tunis, University Tunis El Manar, Tunisia

**Keywords:** *Rhodotorula fungemia*, *Immunocompromised patients*, *Antifungal therapy*

## Abstract

In immunocompromised patients, particularly those with indwelling central venous catheters or hematological disorders, *Rhodotorula spp*. is increasingly implicated in nosocomial infections.

We report a series of four cases of *Rhodotorula mucilaginosa* Fungemia. Two cases involved in adult patients from the intensive care unit with neurological conditions, while the other were pediatric patients with hematological diseases. *Rhodotorula mucilaginosa*. was identified. In vitro antifungal susceptibility testing revealed that fluconazole, voriconazole, and caspofungin exhibited intermediate to complete resistance.

## 1 Introduction

*Rhodotorula* species are ubiquitous basidiomycetous yeasts known for their ability to synthesize pink to red pigments [1]. These environmental yeasts are commonly found in air, soil, and water, as well as on human skin and medical equipment. They can colonize the respiratory, gastrointestinal, and urinary tracts [2]. *Rhodotorula spp*. invasive infections are most common in China but cases have also been reported from other Middle Eastern countries [3,4]. *Rhodotorula* fungemia is emerging as an opportunistic infection, primarily affecting patients with malignancies, neutropenia, or organ transplants and it is frequently associated with patients requiring central venous catheters (CVCs) [5].

The most commonly recovered species from human infections include *R. mucilaginosa, R. glutinis,* and *R. minuta* [5]. We report four cases of *Rhodotorula mucilaginosa* Fungemia.

## 2 Case presentation

### 2.1 Pediatric cases

#### 2.1.1 Case 1

A six-year-old female patient was admitted on day 0 with a diagnosis of idiopathic severe aplastic anemia. Following induction therapy, she underwent an HLA-haploidentical allogeneic hematopoietic stem cell transplantation. For graft-versus-host disease (GVHD) prophylaxis, ciclosporin and methotrexate were administered. In addition, acyclovir was used for cytomegalovirus (CMV) prophylaxis, and granulocyte colony-stimulating factor (G-CSF) was initiated because of prolonged neutropenia.

Primary graft rejection was subsequently confirmed based on chimerism analysis, detection of anti-HLA antibodies, and hypocellular bone marrow findings. The graft rejection was likely associated with the multiple red blood cell and platelet transfusions received during hospitalization, which may have contributed to alloimmunization.

A second hematopoietic stem cell transplantation was planned after a two-month delay to reduce the potential cardiotoxic effects associated with intensive immunosuppressive therapy. During hospitalization, the patient developed several episodes of bacteremia and was treated with broad-spectrum antibiotics. CMV reactivation was detected by real-time PCR using the Rotor-Gene Q platform and was successfully managed.

Voriconazole therapy was initiated following a suspected case of invasive pulmonary aspergillosis, based on positive serum galactomannan antigen testing (index = 1 in two separate serum samples; Platelia Aspergillus Ag, Bio-Rad) and radiological evidence of left basal pneumopathy.

Subsequently, the patient developed polymicrobial septicemia caused by *Pseudomonas luteola* and *Pseudomonas monteilii*, which were isolated from blood cultures and identified using the Vitek system. The infection was successfully treated with appropriate antimicrobial therapy. However, two days before the scheduled second transplant (day +71), the patient developed fever. Blood cultures obtained at day +75 yielded *Rhodotorula mucilaginosa*, identified by Vitek MS with a confidence score of 99.9%.

Antifungal susceptibility testing performed using the E-test® method (bioMérieux, France) showed that the isolate was susceptible to amphotericin B(MICs: 0.25 μg/mL), exhibited intermediate susceptibility to voriconazole (MICs: 2 μg/mL), and demonstrated resistance to fluconazole (MICs ≥ 64 μg/mL) and caspofungin (MICs ≥ 32 μg/mL). Consequently, voriconazole was discontinued on day +76 and replaced with a combination of amphotericin B and caspofungin.

The patient showed a favorable clinical response to treatment. In fact, blood cultures became negative within 24 h after initiation of amphotericin B therapy, with resolution of fever. The duration of fungemia was two days.

#### 2.1.2 Case 2

A nine-year-old male patient was admitted on day 0 for allogeneic hematopoietic stem cell transplantation as treatment for acute B-cell lymphoblastic leukemia. During hospitalization, a central venous catheter was inserted for the administration of conditioning chemotherapy. The patient subsequently developed a bacterial infection caused by *Klebsiella pneumoniae* and *Pseudomonas spp.*, which was treated with piperacillin–tazobactam, teicoplanin, and amikacin.

Despite ongoing antibiotic therapy, the patient developed a persistent high fever on day +20. Empirical antifungal therapy with caspofungin was initiated due to suspicion of an invasive fungal infection (day 20). Blood cultures collected between day +22 and day +23 yielded *Rhodotorula mucilaginosa* identified by Vitek MS score: 99.9%), in 13 separate samples.

Antifungal susceptibility testing performed using the E-test® method (bioMérieux, France) revealed high MICs indicating resistance to fluconazole (MICs ≥ 256 μg/mL), caspofungin (MICs ≥ 32 μg/mL), amphotericin B (MICs = 4 μg/mL), and voriconazole (MICs = 3 μg/mL).

Given these results, antifungal therapy was switched to voriconazole, and the central venous catheter was removed on day +23, one day after confirmation of fungemia. The patient became afebrile on day +24. Follow-up blood cultures obtained after seven days of antifungal therapy were sterile (day +29). Consequently, the exact duration of fungemia could not be accurately determined. The patient's clinical condition progressively improved, resulting in a favorable outcome.

#### 2.1.3 Adult cases

Both patients were adult males aged 27 and 48 years, respectively. They were admitted on the same day (day 0) to the Neurology Department for the management of inflammatory dysimmune neuropathies: autoimmune encephalitis in the first patient and polyradiculoneuropathy in the second. Despite receiving etiology-specific treatments, including corticosteroids and immunotherapy, both patients experienced severe clinical deterioration, with progressive neurological impairment and respiratory failure.

Due to worsening clinical conditions, both patients were transferred to the intensive care unit (ICU), where mechanical ventilation was initiated and central venous catheters (CVCs) were inserted. During their ICU stay, they received broad-spectrum antibiotic therapy for infectious pneumonia for 14 and 20 days, respectively. However, both patients developed persistent fever (≥38 °C) associated with elevated inflammatory markers despite appropriate antibiotic therapy.

Blood cultures were collected on day +14 and day +20, respectively, using Mycosis IC/F bottles and processed in our laboratory. Culture bottles were incubated at 35 °C in the BACTEC system (Becton Dickinson), with positive signals detected after 48–72 h (day +16 and day +23, respectively). Direct microscopic examination revealed spherical to ellipsoidal budding yeasts without hyphal forms. Subcultures performed on Sabouraud dextrose agar and incubated at 27 °C produced smooth, mucoid, orange-pink colonies within 24 h (day +17 and day +24, respectively) (Fig. 1).

**Fig. 1:**
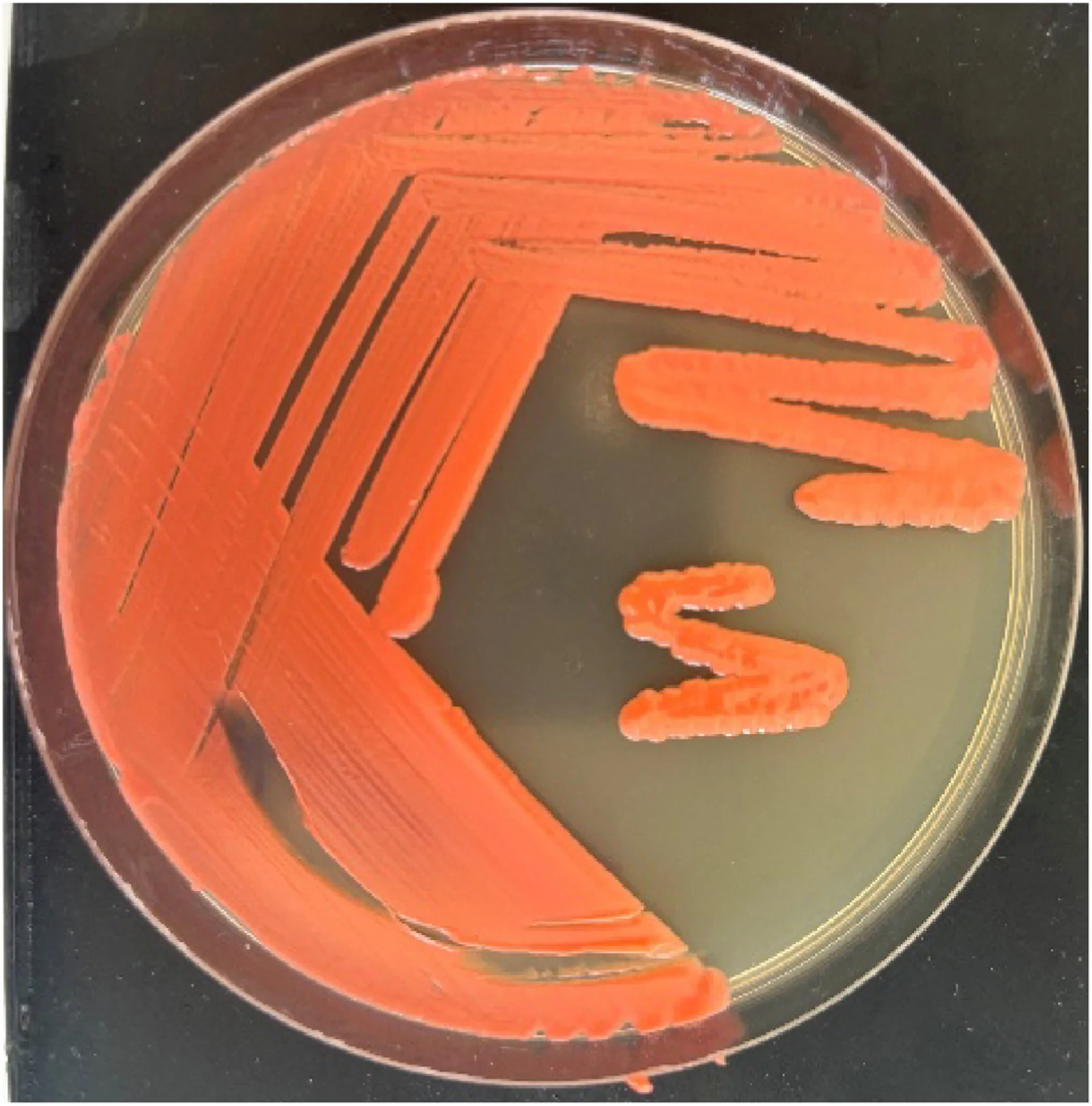
*Rhodotorula spp.* colonies on Sabouraud-Chloramphenicol agar.

Species identification was performed using Vitek MS, which identified *Rhodotorula mucilaginosa* with a confidence score of 99.9%. Urease activity was assessed by inoculating urea-indole medium with a pure colony of *Rhodotorula mucilaginosa*, followed by incubation at 37 °C for 4 h. A color change of the medium from yellow to pink indicated a positive urease reaction.

Antifungal susceptibility testing performed using the E-test® method (bioMérieux, France) demonstrated susceptibility to amphotericin B (MICs = 0.5 μg/ml) but high-level resistance to fluconazole (MICs ≥ 256 μg/mL), MICs ≥ 8 μg/mL), and caspofungin (MICs ≥ 32 μg/mL).

Fluconazole therapy was initiated in both patients upon positivity of blood cultures (day +16 and day +23, respectively). In the first patient, antifungal therapy was switched to amphotericin B on day +19 following the susceptibility results. In contrast, fluconazole therapy was maintained in the second patient due to impaired renal function and the unavailability of liposomal amphotericin B. The central venous catheter was replaced on day +25, two days after fungemia was confirmed.

Despite these interventions, both patients continued to deteriorate clinically, ultimately developing septic shock leading to multiple organ failure and death (day +35 and day +32, respectively). Follow-up blood cultures in the first patient became negative within one day after initiation of amphotericin B therapy, corresponding to a fungemia duration of five days. In contrast, blood cultures in the second patient remained persistently positive until death, with a fungemia duration of ten days.

## 3 Discussion

This report describes four cases of fungemia caused by *Rhodotorula mucilaginosa*. *Rhodotorula* fungemia is defined as the isolation of this yeast from blood cultures [6].

Among the species reported in human infections, *R. mucilaginosa* is the most frequently identified, followed by *R. glutinis* and *R. minuta* [3].

In the present study, *R. mucilaginosa* isolates were identified using MALDI-TOF mass spectrometry (Vitek MS) from pure cultures grown on Sabouraud agar. This technique provides rapid species-level identification with a relatively low cost per test [7].

However, PCR sequencing of the ITS1 and ITS2 regions of ribosomal DNA was not performed, which represents a limitation of the present study. ITS sequencing remains the reference method for definitive species confirmation and taxonomic characterization [7].

This yeast is increasingly recognized as an emerging opportunistic pathogen. The overall incidence of *Rhodotorula* fungemia has been estimated at approximately 0.05 cases per 1000 hospital admissions in Brazil [1]. Although invasive *Rhodotorula* infections have been most frequently reported in China, cases have also been documented in several Middle Eastern countries (Table 1).

**Table 1:** *Rhodotorula* Fungemia cases in Tunisia and Middle Eastern countries.

Country	year	Number of cases	species	Reference
Tunisia	2016	2	*Rhodotorula mucilaginosa*	[8]
Saudi Arabia	1999	1	*Rhodotorula rubra*	[9]
Kuwait	2011	1	*Rhodotorula mucilaginosa*	[10]
Qatar	2021	1	*Rhodotorula mucilaginosa*	[11]

Among the reported cases, the two pediatric patients were admitted for bone marrow transplantation as treatment for hematological diseases (aplastic anemia and acute B-cell lymphoblastic leukemia). The incidence of *Rhodotorula* infections in bone marrow transplant recipients has been estimated at approximately 9.58 cases per 1000 transplants [12]. Furthermore, a systematic review examining the epidemiology and outcomes of *Rhodotorula* infections reported that 67.1% of affected patients had hematologic malignancies. Neutropenia and malignancy are known to significantly increase susceptibility to opportunistic infections [5].

All four patients in this study had undergone invasive medical procedures and received cytotoxic or immunosuppressive treatments, including central venous catheter placement, chemotherapy, corticosteroids, immunotherapy, and broad-spectrum antibiotic therapy. These factors have been identified in several studies as major predisposing conditions for *Rhodotorula* infections [5]. In addition, prophylactic administration of fluconazole has frequently been reported in patients who subsequently develop *Rhodotorula* fungemia. This was not observed in our patients, whereas the six-year-old female patient was receiving voriconazole [13].

Antifungal susceptibility testing was performed on the isolates against commonly used antifungal agents (amphotericin B, fluconazole, voriconazole, and caspofungin) to guide therapeutic decisions. Three of the four isolates showed in vitro susceptibility only to amphotericin B, while the fourth isolate exhibited resistance to all tested antifungal agents.

Azole antifungals were used in the treatment of two patients: fluconazole was administered to the adult patient with renal failure because liposomal amphotericin B was unavailable, and voriconazole was prescribed for the nine-year-old child. However, *Rhodotorula* species are known to exhibit poor susceptibility to azole antifungals, particularly fluconazole, making this drug generally inappropriate for the treatment of *Rhodotorula* infections [14]. In addition, caspofungin was combined with amphotericin B in the treatment of the six-year-old patient. Nevertheless, echinocandins are not considered effective therapeutic options for *Rhodotorula* infections because these species exhibit intrinsic resistance to echinocandins, including caspofungin [15].

The treatment of choice for *Rhodotorula* infections, as recommended by the ESCMID/ECMM guidelines, is amphotericin B [2]. Liposomal amphotericin B, with or without flucytosine, is moderately recommended as first-line therapy. Amphotericin B deoxycholate, with or without flucytosine, is marginally recommended as an alternative first-line option. In contrast, the use of triazoles and echinocandins is discouraged because of the intrinsic resistance of *Rhodotorula* species to these antifungal classes [16].

Removal of the central venous catheter is also strongly recommended [16]. However, in our series this intervention was performed in only two patients, both of whom did not receive amphotericin B, one day after fungemia was confirmed. In fact*, Rhodotorula* species are known to form biofilms on intravascular devices, which may act as a persistent source of infection [14]. Consequently, removal of the central venous catheter has been identified as an important strategy in the management of *Rhodotorula* fungemia and has been associated with improved survival rates [2].

Reported mortality rates associated with *Rhodotorula* fungemia range from 10% to 20% [15]. Although infections caused by *Rhodotorula* species are generally associated with lower mortality than those caused by *Candida* spp., early identification of at-risk patients and prompt initiation of appropriate diagnostic and antifungal therapeutic strategies remain crucial.

In our series, both adult patients died. The duration of fungemia was five and ten days, respectively, which may have been related to delays in the initiation of effective antifungal treatment.

In conclusion, the increasing number of reported cases of Rhodotorula fungemia, together with the high level of intrinsic resistance of this genus to several antifungal agents, highlights the importance of strict adherence to treatment guidelines, prompt removal of intravascular catheters, and systematic follow-up blood cultures to document clearance of fungemia.

## CRediT authorship contribution statement

**Amira Dallali:** Writing – original draft, Investigation, Formal analysis, Data curation, Conceptualization. **Aicha Kallel:** Writing – review & editing, Methodology, Investigation, Data curation, Conceptualization. **Sarra Cheikhrouhou:** Writing – review & editing, Investigation. **Sonia Marouen:** Investigation. **Nour Ben Abdeljelil:** Investigation. **Chokri Kaddour:** Investigation. **Kalthoum Kallel:** Validation, Supervision.

## Ethical Form

The work described has been carried out in accordance with The Code of Ethics of the World Medical Association (Declaration of Helsinki) for experiments involving humans. The research met our institutional definition of a case series and thus institutional research board review was not needed. As we have used anonymized images and we have removed all patient identifiers, consent was not obtained.

## Funding source

All sources of funding should be acknowledged and you should declare any extra funding you have received for academic research of this work. If there are none state ‘there are none’.

## Conflict of interest

Please declare any financial or personal interests that might be potentially viewed to influence the work presented. Interests could include consultancies, honoraria, patent ownership or other. If there are none state ‘there are none’.
